# Clinical Lecture on Croup

**Published:** 1874-12-01

**Authors:** H. Roger


### CLINICAL LECTURE ON CROUP.

By Dr. H. Roger.

*From Obstetrical Journal of Great Britain and Ireland.*

M. ROGER had exhibited at a single clinique a great many more cases of croup than one would see in two or three years of private practice, where the disease is not so common as one might at first be disposed to think. What one often meets with in practice is *false* croup; for example, in Paris there are about one hundred cases per day, while the hospital is the ordinary theatre of *true* croup, and but rarely the refuge of false croup. The reason of this is easy to see; it is because croup without false membranes does not last, or lasts only a few hours, and the little patients, being suddenly attacked in the night, are not sent to the hospital.

Croup, at Paris, is much more frequent than it was thirty or forty years ago; it is true that the population was then less by two-thirds than it is now, for in 1832 (the year of the cholera) the capital contained only 745,000 inhabitants. There exists, however, at the present time, a notable difference between the different kinds of croup; we have only to read the statistics to see that in membranous sore throat one out of every two patients dies, but in croup four out of five. We are, then, naturally impressed when we see so large a number of cases of croup. Apropos of this, M. Roger reminds us that when he was *interne* at the Children's Hospital, under M. Guersant, he saw during a whole year only four or five cases of croup. In the present day tracheotomy is performed about one hundred times a year, and M. Roger has seen even two hundred cases of croup operated on in a single year; at St. Eugenie's Hospital the number is still higher. Considering then, that there are on an average from two to three hundred operations annually in the two hospitals, we may conclude that there are about five hundred cases of croup. Formerly there were very few operations performed, three or four in the year. In the present day, however, the number of cures is greater.

Croup is a contagious disease which operates in epidemics of greater or less gravity, and which presents in different cases differences as to its history, its symptoms, and its form. To illustrate this M. Roger presented six little patients whose cases in more than one particular excited some interest. The first case was that of a little girl suffering from typhoid fever, who had been seized with membranous sore throat, which threatened to become croup; the second was a boy in M. Labric's ward, who was attacked with croup and measles at the same time; then another little girl and another little boy, operated on on the 9th April; in these cases the illness had begun by sore throat which became membranous, followed by croup. Then came a boy who had been operated on on March 31st, and who went on as well as possible. Lastly, a little girl, cured of croup, but who was at the time suffering from pneumonia, which came on during her convalescence.

What does clinical observation teach us? That there is a false croup and a true croup. False croup is simple inflammatory laryngitis, shown by the redness of the laryngeal membrane; in the more severe cases it is complicated by œdema, but *there never exist what are called false membranes.*

What then is true croup? We must begin by explaining certain words. It has been said that there are two kinds of croup, *inflammatory* or *strangulatory*, and *common* croup. This last is not a satisfactory name, and is inexplicable by any clinical observation. It is, in fact, the same with croup as with scarlet fever, for example, in which we admit two degrees but not two kinds of scarlet fever. In croup, says M. Roger, the degree of poisoning is in proportion to the length of the false membranes.

*Localized Croup* has also been called *herpetic croup*. This word gives an incorrect idea of the thing; the mistake consists in trying to make a variety of it; it would be better to call it a *mild* form, in opposition to the *generalized* or *malignant* form.

Croup is very variable also in its mode of generalization, whether there exists a laryngeal, or pharyngeal, or bronchial lesion; sometimes there is also coryza or sore throat. This croup has been called *infectious* or *toxic*. What constitutes the gravity of the disease is not, properly speaking, the false membrane, for the operation does away at once with that obstacle, but its reproduction and propagation.

Croup may be *primitive* or *secondary*. Secondary croup occurs in the course of a disease already existing, and has evidently a pathological connexion with the first illness. It may succeed *measles*, in which the laryngeal-bronchitic element has been predominant, and in which children are sometimes attacked by severe laryngobronchitis, which may become transformed into secondary croup. In the same way, in scarlet fever, there is a scarlet-fever sore throat, membranous, which sometimes, though not often (as Trosseau and G. See have observed), by invading the larynx, may become secondary croup. In the case of the little patient in M. Labric's ward it is not one of secondary croup, because the croup and the measles appeared at the same time: the false membrane was first seen March 30th, the croup, properly speaking, on the 8th April, measles having shown itself on the 4th April. How shall we name the other forms of croup, which appear as complications; for example, that in typhoid fever, where no pathological relation can be said to exist between the two diseases? In these cases the croup is quite accidental.

It remains, then, to find out what epithet should be applied to the croup which succeeds severe membranous sore throat, and which is, in fact, characterized by the extension of the false membranes to the larynx. M. Roger calls it *successive* and not *secondary*. The same thing may be said of the croup, which begins by a membranous coryza, the plastic productions invade the larynx equally in this case, and it is again the same process of intoxication.

*Etiology.*—Croup is a *specific* and contagious disease. This definition justifies all the causes attributed to the disease. Forty years ago *cold* was said to have a great share in its production; that notion is true in so far that in the cold season croup often assumes a more serious character, being complicated with inflammation of the respiratory organs. Thus, at the present moment, (April,) instead of twenty-five per cent. as the proportion of cures, we find thirty and even forty per cent. Season and climate, however, have no absolute influence; as examples of this we may cite the epidemics which took place in the seventeenth century in Spain and Italy, and the Syrian ulcer described by Aretee, of Cappadocia. (In Cappadocia the thermometer marks 38° C. in the shade.) If misery has any influence in determining croup, it is owing to the crowding, the packing up, if one may say so, which results from it, and which constitute the most favorable conditions for contagion. *Sex* has no influence on croup. *Age*, on the contrary, has certain privileges in this respect. Although a person of any age may have croup, children are the most often attacked, and that principally between three and four years of age. In second childhood croup is rare; and it is quite the exception with adults.

As for the *contagiousness* of croup, it is very great and only too evident, and more evident in town than in hospital. Let us take as an example one personal . experience among a thousand, related by M. Roger. He was called to Boulogne-sur-Mer to see a child attacked by croup, but arrived an hour too late, as the child was dead. The ordinary medical attendant, a very able practitioner, former *interne* at the Children's Hospital, had taken the precaution of sending the other two children to Paris. The father also returned to Paris, and, notwithstanding the time (fourteen days) that had elapsed since the death of the first child, the other two children were attacked with the disease, and carried off in a few hours. Have we not had the grief of numbering also amongits victims some of our illustrious masters—Gillette, Valleix, the son of M. 'Blache ? to these names, whom we may designate the children's doctors, respect imposes on us the duty of adding another compatriot, the son of M. de Salle, an esteemed practitioner of Chalon-sur-Marne, who died a victim to his devotedness. Mothers are not exempt from the membranous sore throat and the croup of their children; after them come the nurses; lastly, more rarely it is true, the fathers take the disease.

*Forms and Symptoms.*—Croup presents three forms for consideration—the mild form; the *medium* form, which is the most common; and lastly, the *toxic* form—toxic from the onset, or rather in which the poison is evident.

M. Roger, in studying the symptoms of this affection, has no intention to describe them completely, nor to enter into every detail of the symptoms, but merely to speak of the more salient points and to dwell only upon those which are of importance in a clinical point of view. It is an easy matter to recognize mild croup from the grouping of the symptoms; it is the same in this respect with toxic croup, as the phenomena of poisoning are very recognizable: medium croup, on the contrary, offers certain difficulties, certain snares which are to be avoided, and of which it is well to be forewarned.

It is all-important to distinguish with care the *first period*—that is to say, the *anginous period*, for croup suddenly setting in is rare. Bretonneau used to say, very rare; but the proportion which he has set forth, one in twenty or thirty, is exaggerated. We would give the ratio as one or two in ten; this croup commences in the larynx, especially if it succeeds measles; one can understand that in this case the poison has so much the greater hold, as the mucous membrane is already affected.

The anginous period is almost invariable, and is characterized by the usual phenomena of sore-throat: *a little pain* in the throat (it is necessary to bear in mind that amongst children *sore throat is not painful*), some *redness* and *cough*, and a slight *dyspnœa*. This period is, so to say, latent, and passes in general unperceived by the patients. Sometimes one discovers *false membranes*, which are then situated on the internal face of one or both tonsils, and which consist of patches more or less extensive, which are to be distinguished from the pultaceous or caseous exudation of ordinary sore throat or tonsilitis. What puts one on the track of the disease is *adenitis*: the submaxillary glands, those at the 'angle of the lower jaw, and even those of the lateral and posterior cervical region are tumefied. The cough presents nothing particular, it is the cough of simple bronchitis. This period lasts two or three days.

Then come the phenomena of the *laryngeal period*; the poison of the throat is at the larynx. An *attack of suffocation* serves as prelude, and makes us think of croup. The *cough* is *hoarse*, *broken*, a little *rough* and metallic.

The *voice* is *suppressed almost suddenly* (a characteristic sign). The *respiration* gives some important characters of recognition: it is *slow, pain-*

*ful;* the chest heaves, the nostrils dilate, there is, in a word, *forcing* of the breath. A sign which is of great value is *noisy respirations*, audible at a distance, rough, at first intermittent, and which the change of position causes to cease, but continues more and more, and which exists no longer in inspiration only, but also in expiration. This is what is called *sawing laryngeal wheezing* (*sifflement larynge serratique*) which becomes more and more manifest in the proportion in which the disease makes progress. Stethoscopic signs are absent; auscultation of the larynx elicits nothing else but a snoring; and there are, perhaps, few practitioners who have heard the *flapping sound* (*bruit de drapeau*) which M. Barth points out; M. Roger, for his part, has never heard it. In *auscultation of the chest* it is difficult to hear the minute vesicular sound, which is almost totally extinguished by the laryngeal snore. As for the rest, the air reaches the lungs in small quantity, and seems to pass into a contracted orifice.

There are tolerablv simple means of distinguishing the true from the false croup. Thus, croup never commences all at once, but after two, three, or four days of prodromata. Œdematous or erythematous laryngitis on the contrary, commences directly with laryngeal symptoms; between midnight and one o'clock in the morning the cough resembles a sort of barking, the voice is retained, and the respiration is free in the intervals of the attacks.. Sometimes this laryngitis is more intense, and the attacks become more continuous, the fever is considerable, but the voice is not completely extinguished as in the true croup. Finally, by auscultation, one cannot perceive the absence of the vesicular murmur.

What are *diseases which can still simulate croup?*

1. *Œdema of the larynx;* but it is exceedingly rare amongst children, and by the examination of the superior part of the arytenio-epiglottic folds all doubt is removed

2. The introduction of *foreign bodies*, such as a cherry-stone, a bean, &c.: but there is no fever; besides, recollection comes in aid, as well as the other signs of which we have spoken.

3. *Retro-pharyngeal abscess*, which gives rise to these symptoms—cough, fever, difficulty of respiration, suffocation; but the examination of the throat suffices to put an end to all idea of croup.

